# Prevalence and factors associated with food intake difficulties among residents with dementia

**DOI:** 10.1371/journal.pone.0171770

**Published:** 2017-02-22

**Authors:** Chia-Chi Chang, Yu-Fang Lin, Chia-Hui Chiu, Yuan-Mei Liao, Mu-Hsing Ho, Yen-Kuang Lin, Kuei-Ru Chou, Megan F. Liu

**Affiliations:** 1 School of Gerontology Health Management, College of Nursing, Taipei Medical University, Taipei, Taiwan; 2 School of Nursing, College of Nursing, Taipei Medical University, Taipei, Taiwan; 3 Center of General Education, Taipei Medical University, Taipei, Taiwan; 4 Biostatistics Research Center, Taipei Medical University, Taipei, Taiwan; University of Brescia, ITALY

## Abstract

**Background:**

Few studies have examined the prevalence of food intake difficulties and their associated factors among residents with dementia in long-term care facilities in Taiwan. The purpose of the study was to identify the best cutoff point for the Chinese Feeding Difficulty Index (Ch-FDI), which evaluates the prevalence of food intake difficulties and recognizes factors associated with eating behaviors in residents with dementia.

**Methods and findings:**

A cross-sectional design was adopted. In total, 213 residents with dementia in long-term care facilities in Taiwan were recruited and participated in this study. The prevalence rate of food intake difficulties as measured by the Chinese Feeding Difficulty Index (Ch-FDI) was 44.6%. Factors associated with food intake difficulties during lunch were the duration of institutionalization (beta = 0.176), the level of activities of daily living-feeding (ADL-Q1) (beta = -0.235), and the length of the eating time (beta = 0.416). Associated factors during dinner were the illuminance level (beta = -0.204), sound volume level (beta = 0.187), ADL-Q1 (beta = -0.177), and eating time (beta = 0.395).

**Conclusions:**

Food intake difficulties may potentially be associated with multiple factors including physical function and the dining environment according to the 45% prevalence rate among dementia residents in long-term care facilities.

## Introduction

As dementia progresses, food intake difficulties will occur, and about 50% of persons with dementia will have such difficulties within 8 years of disease onset [1]; as people near the advanced stage, a majority will suffer the same experiences [2]. There are two solutions when this inevitable situation occurs, continued hand feeding and tube feeding [3]. There is insufficient evidence supporting the benefits of using tubes for feeding persons with dementia [4], and hand feeding allows persons with dementia to enjoy basic food tasting and caregiver socialization [3]. Various situations, which include clamping the mouth shut, delayed swallowing, and refusing to eat, contribute to food intake difficulties when hand-feeding persons with dementia [5].

Food intake difficulties can lead to high risks of dehydration, weight loss, malnutrition, and ultimately death for residents with dementia [6]. In long-term care facilities, weight loss and malnutrition are important quality indexes [7,8]. There are multifaceted factors related to food intake difficulties including physiological and psychological factors in an individual and on a broader sociocultural level [9,10]. Physiological factors comprise motor and visual impairments which might affect the management of eating utensils; the dental situation, oral hygiene, and dysphagia can further influence chewing and swallowing [8,11–14]. Depression is the most common comorbid disease among persons with dementia [15]. Dementia’s cognition and attention impairment, which are pivotally important for older adults to begin and/or complete food intake tasks, will cause food intake difficulties. As for socioculturally related factors, a loose-seated dining environment with a relaxed atmosphere for caregivers to sit with persons with dementia during food intake is crucial [5,16,17].

The prevalence of dementia in Taiwan is 61.8% in assisted living facilities and 64.5% in nursing homes [18]. Given the high prevalence of dementia in long-term care facilities, there are few studies examining the prevalence of food intake difficulties in Taiwan [19]. This study is unique because it included illuminance and sound volume levels of the dining area in order to assess environmental aspects of the food intake process. In addition, the disease trajectory varies among individuals, and thus signs and symptoms of people with dementia may differ during the course of the disease. These differences contribute to various food intake difficulties in people with dementia. Furthermore, some people with dementia experience sundowning syndrome [20], and consequently it is logical to assume distinct situations at different mealtimes. Therefore, it is crucial to understand the prevalence of and factors associated with food intake difficulties at different mealtimes, in order to provide appropriate interventions to promote mealtime quality.

Almost half of residents with dementia in long-term care facilities in Taiwan have food intake difficulties.[19] With the current staff and resident ratio, which is one nurse for every 15 residents and one nursing assistant for every five residents [21], it is hard for staff to assist during meals, not to mention that half of residents may have difficulties such as getting food, being distracted, refusing food, and experiencing motor difficulties [22]. While cognition and activities of daily living (ADLs) are not amenable to change, the dining environment including the illuminance and sound volume are objective and modifiable. Moreover, longer eating times are easy to observe and can serve as an obvious signal for nurses and nursing assistants to be aware of those who may be suffering from difficulties with food intake.

The purposes of this study were to 1) identify the best cutoff point for the Chinese Feeding Difficulty Index (Ch-FDI) and evaluate the prevalence of food intake difficulties; and 2) recognize factors associated with eating behaviors in residents with dementia.

## Methods

### Design

This study had a cross-sectional study design. Research sites and participants were selected based on a purposeful sampling strategy.

### Setting and participants

The long-term care facilities selected in this study were based on establishment standards and evaluation grades in Taiwan. We recruited large-sized (100 beds or more) [23] facilities in northern Taiwan with an evaluation grade of higher than A (The evaluation results were marked as: excellent, grades A, B, C, and D) [24]. In the period of our study, there were 222 long-term care facilities in northern Taiwan (including Keelung City, New Taipei City, and Taipei City), and residents with dementia were conveniently sampled and visited at eight of these long-term care facilities (3.6%).

Participants were enrolled according to the following criteria: 1) aged ≥60 years; 2) had been diagnosed with dementia; 3) were receiving oral feeding with food intake; and 4) were unable to complete a meal by oneself or needed help with food intake as identified by a nursing assistant. In addition, residents who were in a coma or using artificial feeding were excluded. We needed to recruit at least 185 people with dementia based on a medium effect size of 0.15 according to Cohen (1988) for the *F*-test [25], and a power of 0.95 with an alpha of 0.05 using G*power 3.0.1.10 software, as well as an estimated attrition rate of 10%~20%.

### Data collection

We interviewed a resident or caregiver in order to obtain data on demographics (such as gender, age, duration of dementia, duration of institutionalization, ethnicity, education level, marital status, number of diseases, and the number of medications), cognition, independence in ADLs, and depression status. Moreover, we measured the dining area environment including the illuminance and sound volume, amount of food intake, and length of eating time; while we observed the interaction between residents and caregivers as well as residents’ food intake difficulties. Finally, we reviewed medical records in regards to height and weight for calculating the body-mass index (BMI in kg/m^2^).

#### Cognition

The Mini Mental Status Examination (MMSE) is one of the most frequently used tests for screening and monitoring cognitive impairments in older adults. The MMSE has several domains, including orientation to time and place, registration of three objects, attention to serial subtraction, recall of three objects, language, and so on, which are summed to a total score of 30 points with greater impairment resulting in lower scores [26]. The MMSE has been validated in ethnic Chinese societies and used extensively to screen older individuals for cognitive deficits [27–30]. As for illiterate elders, we read item 25 “Close your eyes” to them as suggested by the Taiwan Dementia Society MMSE manual [31].

#### Independence in ADLs

The Barthel index consists of 10 items and was used to examine independence in the following aspects of ADLs: feeding, bathing, grooming, dressing, excavating the bowels, controlling the bladder, using the toilet, transferring, mobility, and navigating stairs. The total score ranges 0~100 with greater dependence resulting in lower scores [32]. The Chinese version of the Barthel index has been widely used in older populations [33], and was confirmed to be a psychometrically sound instrument [34]. ADL question number one (ADL-Q1) was included as an independent variable in the analysis due to its focus of assessing feeding independence.

#### Depression status

The Geriatric Depression Scale-Short Form (GDS-S) consists of 15 "yes/no" items and was developed to screen for depression in older adults, with a more-severe level of depression resulting in a higher score [35]. It was established to have satisfactory psychometric qualities [36]. The Chinese version of the Geriatric Depression Scale-Short Form (GDS-S) was developed and has been widely used in Chinese-speaking societies [28]. Residents with severe dementia who could not respond to GDS-S data on depression status were recorded as missing.

#### Interactions with caregivers

Interactions with caregivers were measured at lunch and dinnertime while people with dementia were being assisted with feeding. This parameter was recorded as "yes" or "no", and total numbers of verbal (talked to others) and non-verbal (such as eye contact) forms of communication with caregivers were recorded.

#### Dining area environment

The illuminance and sound volume levels in the dining area were measured with a digital light meter Tecpel 536 (lux) and a sound level meter Tecpel 331 (dB) during lunch and dinnertime while the food intake process was being measured. Moreover, the source of light (natural light or artificial light or both) and sound (from TVs, radios, or talking noise) in each resident’s dining environment were also assessed by a research assistant. According to the manufacturer of Tecpel 536, 100~200, 300~750, and 750~1000 lux are the desired illuminances of a hospital ward, medical examination room or operating room, and emergency room, respectively. As for the sound, 30~80, 50~100, and 80–130 dB were classified as low, medium, and high volumes, respectively.

#### Food intake difficulties

Food intake difficulties were simultaneously measured using the Chinese Feeding Difficulty Index (Ch-FDI) and the Edinburgh Feeding Evaluation in Dementia (EdFED) scales. Six research assistants (RAs) were trained to collect data and evaluate food intake difficulties among residents with dementia. We used video which recorded ten residents’ food intake behaviors, and each RA had to rate the food intake difficulties in order to build up reliability. Inter-rater reliability was calculated, and the mean agreement rate of the six RAs on the ten residents was 0.87, and the average agreement rate of each item ranged 0.55~0.98.

The EdFED was the first published scale for measuring food intake difficulties in people with dementia, and it was confirmed to be both a practical and psychometrically sound instrument. Chang’s (2012) study suggested 5 to be the cutoff point of the EdFED [37]. Moreover, this study indicated that some behaviors of people with dementia during the feeding process were not captured by the EdFED. Therefore, the Ch-FDI scale was developed in order to more specifically observe food intake difficulties of people with dementia in order to design appropriate interventions.

The Ch-FDI has 19 items, and it is administered by observing the amount of food offering influenced by certain aspects such as difficulty getting food, being distracted, refusing food, and experiencing motor difficulties at each meal. The severity of each item was recorded as the number of times and converted to points as 0 times (0 point), 1 or 2 times (1 point), 3~5 times (2 points), and more than 6 times (3 points). The total score of the Ch-FDI ranges 0~57 points, with greater feeding difficulty resulting in a higher score. The Ch-FDI has fair psychometric properties with an internal consistency Cronbach's alpha of 0.61 and a test-retest coefficient of 0.85. The Ch-FDI was developed to measure food intake difficulties in residents with dementia in long-term care facilities [19]. In this study, food intake difficulties were measured during both lunch and dinner, and we also had an overall score of the Ch-FDI which was the average score of Ch-FDI at lunch and dinner.

#### Amounts of food consumed

Amounts of food consumed (in grams) were measured by weighing the food plus the tray before and after a meal. To estimate the food lost, the amount of food that could be retrieved was weighed. Clothing and napkins were weighed before and after feeding to estimate the amount of food contained on those articles. In addition, the types of food (regular, soft, semi-liquid, and liquid) and types of utensils (spoon, chopsticks, and hand) were recorded in order to understand routine eating behaviors.

#### The length of the eating time

The eating time was recorded as the duration of the tray being placed in front of a resident to the tray being taken away. Specifically, in terms of the operational definition, time recording began when a nursing assistant placed a meal on the resident’s table and the resident began to eat; time recording ended when the nursing assistant considered the resident to have finished the meal and removed the tray. Nursing assistants decided when to remove the tray based on their experience, while research assistants recorded eating times through direct observations without intervening.

#### Body-Mass Index (BMI)

The BMI of residents was determined by reviewing their medical charts a week before data collection, which included the weight and height of residents in order to compute their BMI as [weight in kg]/[height in m]^2^) (S1 File).

#### Ethical considerations

Institutional review board approval of this study was obtained from the Human Subject Committee of Taipei Medical University (approval no.: P970331), and residents with dementia who met the inclusion criteria were recruited. First, we obtained agreement from the long-term care facilities, and then we asked for the informed consent of those residents as well as their caregiver proxies, during the process of which, the purposes and the process of the study were clearly explained. After acquiring informed consent from residents with dementia and their proxies, data collection was based on the instruments which were designed for this study by observing people with dementia during lunch and dinner.

#### Data analysis

All data were analyzed with the computerized statistical software, IBM SPSS statistics 22.0. Descriptive statistics were performed including means, standard deviations (SDs), frequencies, and percentages. We used a receiver operating characteristic (ROC) curve analysis in order to identify the best cutoff point for the Ch-FDI. The reference was the EdFED as the gold standard, which classifies food intake difficulties in people whose scores exceeded 5 points as suggested by a previous study [37]. Moreover, the ROC curve analysis also provided the area under the curve (AUC) of the Ch-FDI, which demonstrated the accuracy of this scale. In general, the AUC from the ROC curve exceeded 0.7 which is considered acceptable discrimination [38]. The highest Youden’s index (sensitivity + specificity − 1) provided the best cutoff point, and we then accordingly calculated the prevalences of EdFED and Ch-FDI [39]. A general linear model (GLM) was used as an inferential statistical strategy, and Pearson’s correlations were used to determine relationships between all independent variables and food intake difficulties. Furthermore, stepwise multiple regressions were used to identify potential variables associated with food intake difficulties at both lunch and dinner.

## Results

### Participant characteristics

In total, 221 residents with dementia agreed to participate in this study, in the process, eight residents withdrew due to unavailability, including relocation, admission to the hospital, or death. Therefore, 213 residents were included with a response rate of 96.4%.

In total, there were 213 participants in this study. More than half of the participants were female (57.3%, *n* = 122) and the mean age was 82.6 (SD = 6.7) years. While the mean length of time with a diagnosis of dementia was 2.5 (SD = 2.4) years, the average duration of living in an institution was 2.6 (SD = 2.3) years, and the average number of other diseases was 2.4 (SD = 1.1), and number of medications was 4.0 (SD = 1.5). Mean scores of the MMSE, ADL, ADL-Q1, and GDS-S were 8.9 (SD = 8.2), 40.4 (SD = 33.7), 6.06 (SD = 4.1), and 3.5 (SD = 2.9), respectively (Table 1). The mean level of illuminance was 474.0 (SD = 417.3) lux, and the mean sound volume in the dining rooms was 60.3 (SD = 3.8) dB. Around 60% of participants engaged in verbal or non-verbal communication during mealtimes. The average eating time was 20.6 (SD = 15.1) min, and average food intake was 506.2 (SD = 150.5) g. The mean score of the EdFED was 4.9 (SD = 3.9), and that of the Ch-FDI was 4.0 (SD = 3.5). The mean BMI was 21.5 (SD = 3.8) kg/m^2^, and 21.6% of residents (*n* = 46) had a lower BMI than the 18.5 kg/m^2^ defined by WHO recommendations as being malnourished.

**Table 1:** Characteristics of residents and their dining environment (*N* = 213).

Variable	Mean ± SD	*n* (%)
Gender		
Male		91 (42.7)
Female		122 (57.3)
Age (years)	82.6 ± 6.7	
Duration of dementia (years)	2.5 ± 2.4	
Duration of institutionalization (years)	2.6 ± 2.3	
Ethnicity		
Minnanese (Taiwanese)		129 (60.6)
Hakka		8 (3.8)
Mainlander		74 (34.7)
Others		2 (0.9)
Education		
Illiterate		53 (24.9)
Informal education		10 (4.7)
Elementary		65 (30.5)
Junior-high		23 (10.8)
Senior-high		29 (13.6)
College		32 (15.0)
Masters or above		1 (0.5)
Marital Status		
Single		16 (7.5)
Married		72 (34.0)
Divorced		9 (4.2)
Widowed		115 (54.2)
Number of diseases		2.4 (1.1)
Number of medications		4.0 (1.5)
MMSE (score)	8.9 ± 8.2	
ADL (score)	40.4 ± 33.7	
ADL-Q1 (score)	6.1 ± 4.1	
GDS-S (score) (*N* = 135)	3.5 ± 2.9	
Body-mass index (kg/m^2^)	21.5 ± 3.8	
Eating time (min)	20.6 ± 15.1	
Amount of food consumed (g)	506.2 ± 150.5	
Level of illuminance (lux)	474.0 ± 417.3	
Level of illuminance (lux)-Lunch	550.1 ± 646.7	
Level of illuminance (lux)-Dinner	398.0 ± 290.2	
Level of sound volume (dB)	60.3 ± 3.8	
Level of sound volume (dB)-Lunch	60.0 ± 4.2	
Level of sound volume (dB)-Dinner	60.6 ± 4.4	
EdFED	4.9 ± 3.9	
Ch-FDI	4.0 ± 3.5	
Ch-FDI-Lunch	4.2 ± 3.9	
Ch-FDI-Dinner	3.9 ± 3.8	

Abbreviations: MMSE, Mini-Mental Status Examination; ADL, independence in activities of daily living; ADL-Q1, independence in activities of daily living-question 1 (feeding); GDS-S, Geriatric Depression Scale-Short form; EdFED, Edinburgh Feeding Evaluation in Dementia; Ch-FDI, Chinese Feeding Difficulty Index.

### Prevalence rates of food intake difficulties and associated factors

**Fig 1:**
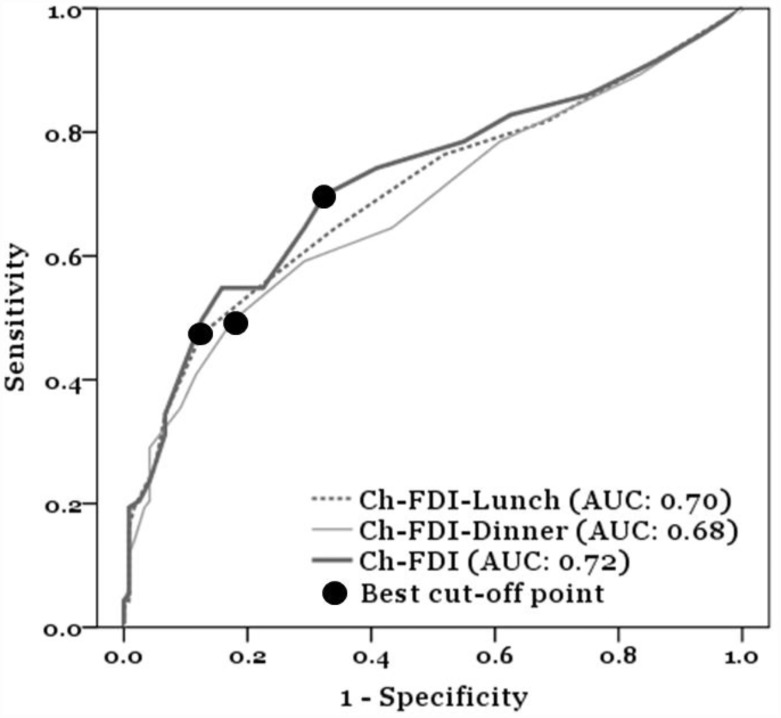
Receiver Operating Characteristic (ROC) curve for the Ch-FDI. Notes: Ch-FDI’s AUC was 0.721(SE = 0.037, CI = 0.649–0.792) which provided acceptable discrimination; Ch-FDI’s AUC at lunch time and dinner time were 0.702 (SE = 0.037, CI = 0.629–0.775), and 0.68 (SE = 0.038, CI = 0.606–0.755). Abbreviations: Ch-FDI, Chinese feeding difficulty index; AUC, area under the curve.

The prevalence of food intake difficulties according to the EdFED (EdFED≥5) was 43.66%, using a cutoff point of 5 based on Chang’s (2012) study [37]. The ROC curve analysis according to the highest Youden’s index showed results of 0.348 and 0.32, indicating that the best cutoff point for lunch was 5 points, and that for dinner was 4 points. The AUCs were 0.702 (SE = 0.037, CI = 0.629~0.775) at lunchtime and 0.68 (SE = 0.038, CI = 0.606~0.755) at dinnertime, giving a good indication of the Ch-FDI. In a subsequent analysis, prevalences of food intake difficulties according to the Ch-FDI at lunchtime (Ch-FDI-Lunch ≥5) and dinnertime (Ch-FDI-Dinner ≥4) were 36.2% and 42.3%, respectively. In order to better understand the overall prevalence of food intake difficulties according to the Ch-FDI, we repeated the above analysis strategy using an ROC curve analysis and Youden’s index which showed a result of 0.374, with an AUC of 0.721 (SE = 0.037, CI = 0.649~0.792) which was acceptable and validated the Ch-FDI’s cutoff point. Accordingly, an optimal cutoff point of 4 was determined, and thus the overall prevalence of food intake difficulties was 44.6% (Ch-FDI ≥4) (Fig 1).

Food intake difficulties, as measured by the Ch-FDI during lunchtime, were significantly correlated with cognition (*r* = -0.201, *p* = 0.003), independence in ADLs (*r* = -0.181, *p* = 0.008), the ADL-Q1 (*r* = -0.231, *p* = 0.001), BMI (*r* = -0.137, *p* = 0.046), and length of eating time (*r* = 0.391, *p*<0.001) (Table 2). Using a multiple regression to examine the associated factors of Ch-FDI scores during lunchtime, 24.0% of the variance in food intake difficulties was explained by the duration of institutionalization (beta = 0.176), the ADL-Q1 (beta = -0.235), and eating time (beta = 0.416), after adjusting for age, gender, cognition, duration of time with a diagnosis of dementia, numbers of diseases and medications, illuminance and sound volume levels of the dining area, and social interactions (Table 3). Furthermore, food intake difficulties as measured by the Ch-FDI during dinnertime were significantly correlated with cognition (*r* = -0.167, *p* = 0.015) ADL-Q1 (*r* = -0.143, *p* = 0.037), the illuminance level (*r* = -.0193, *p* = 0.005), sound volume level (*r* = 0.146, *p* = 0.033), and eating time (*r* = 0.339, *p*<0.001). Factors associated with Ch-FDI scores during dinnertime were the illuminance level (beta = -0.204), sound volume level (beta = 0.187), ADL-Q1 (beta = -0.177), and eating time (beta = 0.395). These factors explained 20.7% of the variance in food intake difficulties after adjusting for age, gender, duration of dementia, duration of institutionalization, numbers of diseases and medicines, and amount of food consumed (Table 4).

**Table 2:** Correlations between independent variables and the Ch-FDI (*N* = 213).

Independent variables	FDI-Lunch	FDI-Dinner
MMSE	-0.201**	-0.167*
ADL	-0.181**	-0.130
ADL-Q1	-0.230**	-0.143*
Body-mass index	-0.137*	-0.059
Duration of institutionalization	0.130	0.017
Eating time (minutes)	0.391***	0.339***
Level of illuminance	-0.076	-0.193**
Level of sound volume	0.049	0.146*

Notes: Pearson correlation coefficients and *p* values for the variables examined.

* *p*<0.05,

** *p*<0.01,

*** *p*<0.001.

Abbreviations: MMSE, Mini-Mental Status Examination; ADL, independence in activities of daily living; ADL-Q1, independence in activities of daily living-question 1 (feeding).

**Table 3:** Multiple regression analysis for factors associated with the Ch-FDI-Lunch (*N* = 213).

Variables	B	SE	β	*t*	VIF
Constant	1.504	5.444		.276	
Age	-0.039	0.037	-0.067	-1.052	1.123
Gender (ref: female)	-0.076	0.504	-0.010	-0.150	1.164
Number of disease	-0.033	0.227	-0.010	-0.147	1.200
Number of medication	0.101	0.170	0.039	0.592	1.229
Duration of dementia	-0.010	0.012	-0.074	-0.835	2.152
Duration of institutionalization	0.025	0.012	0.176	1.990*	2.165
Level of illuminance	-0.001	0.000	-0.084	-1.352	1.083
Level of sound volume	0.106	0.058	0.117	1.841	1.113
MMSE	-0.037	0.034	-0.078	-1.086	1.420
ADL-Q1	-0.220	0.068	-0.235	-3.241***	1.460
Body-mass index	-0.085	0.066	-0.084	-1.289	1.178
Interactions with caregivers	0.415	0.268	0.105	1.549	1.268
Eating time	0.094	0.014	0.416	6.507***	1.134
Regression model	*R*^*2*^ = 0.287 *adjusted R*^*2*^ = 0.240				
	*F*(14,198) = 6.135 (*p*<0.001)				

Notes:

*p < .05,

**p < .01,

***p < .001

Abbreviations: MMSE, Mini-Mental Status Examination; ADL-Q1, independence in activities of daily living-question 1 (feeding).

**Table 4:** Multiple regression analysis of factors associated with the Ch-FDI-Dinner (*N* = 213).

Variables	B	SE	β	*t*	VIF
Constant	-6.450	5.468		-1.180	
Age	0.005	0.037	0.009	0.145	1.117
Gender (ref: female)	0.076	0.515	0.010	0.147	1.195
Number of disease	0.059	0.228	0.017	0.257	1.199
Number of medication	0.063	0.173	0.025	0.364	1.263
Duration of dementia	-0.003	0.012	-0.020	-0.216	2.211
Duration of institutionalization	-0.001	0.013	-0.006	-0.064	2.253
Level of illuminance	-0.003	0.001	-0.204	-3.214**	1.075
Level of sound volume	0.162	0.057	0.187	2.833**	1.155
MMSE	-0.059	0.034	-0.127	-1.740	1.409
ADL-Q1	-0.164	0.068	-0.177	-2.398*	1.447
Body-mass index	0.022	0.067	0.022	0.328	1.194
Interactions with caregivers	0.509	0.287	0.116	1.775	1.140
Amount of food intake	-0.001	0.002	-0.034	-0.530	1.126
Eating time	0.101	0.016	0.395	6.160***	1.094
Regression model	*R*^*2*^ = 0.260 *adjusted R*^*2*^ = 0.207				
	*F*(14,198) = 4.932 (*p*<0.001)				

Notes:

* *p*<0.05,

** *p*<0.01,

*** *p*<0.001.

Abbreviations: MMSE, Mini-Mental Status Examination; ADL-Q1, independence in activities of daily living-question 1 (feeding).

## Discussion

This study found that residents with dementia in Taiwan had an approximately 45% prevalence rate of food intake difficulties (43.66% measured by the EdFED and 44.6% measured by the Ch-FDI). Results of this study are similar to those of Lin (2010) who revealed a prevalence of 30.7% with a low amount of food intake at meals among residents with dementia [40]. However, in Lin’s study (2010) the EdFED was used to measure food intake difficulties, and we used both the EdFED and Ch-FDI instruments. The benefit of including the Ch-FDI to assess food intake difficulties was to better identify eating behaviors, such as getting food into the mouth, chewing, swallowing, and paying attention to the task of eating [22,40]. Moreover, in our study, we included social and environmental factors which are modifiable and can provide better clinical implications.

The only psychological factor included in this study was the depression status, and it had no correlation with food intake difficulties at either lunch or dinner in this study. This may have been due to the fact that approximately 40% of the residents were unable to complete the GDS-S due to their severe dementia.

Our study supports previous findings that showed a significant relationship between the environment and food intake difficulties, including eating time, and illuminance and sound volume levels which portray certain aspects of the physical and social environment [41]. However, while Slaughter and colleagues (2011) concluded that half of the food intake problems might not be due to dementia or its comorbidities [41], our study found that the MMSE was modestly negatively associated with the Ch-FDI at both lunch and dinner. In other words, lower cognitive function may lead to greater food intake difficulties, while causalities were not supported in our study.

We considered the ADL-Q1 in addition to ADLs due to its focus on the ability to feed oneself. This inclusion was in accordance with results of this study which only exhibited a correlation between ADLs and food intake difficulty at lunchtime. Yet the ADL-Q1 was correlated with food intake difficulties at both lunch and dinner. Both the ADL and ADL-Q1 had modest negative correlations with food intake difficulties, which demonstrated that patients with greater dependence or greater feeding dependence had more food intake difficulties. According to the results of the logistic regression, ADL-Q1 was also associated with minimal food intake difficulties at dinnertime (adjusted odds ratio (AOR) = 0.90, 95% CI: 0.83~0.97).

The Ch-FDI was administered by observing the entire mealtime in an attempt to capture all of the problems during the eating process. Eating time was positively correlated with the Ch-FDI. In other words, a longer eating time might be indicative of a higher level of food intake difficulties. According to the results of the logistic regression, eating time was associated with food intake difficulties at both lunch (AOR = 1.070, 95% CI: 1.04~1.10) and dinner (AOR = 1.10, 95% CI: 1.06~1.14). Eating time had the greatest significance among all variables in this study, demonstrating it to be a major factor in food intake difficulties at both lunch and dinner among residents with dementia. Training/education programs were found to decrease food intake difficulties in older adults with dementia [9]. Longer eating times are easy to observe, and thus special attention needs to be paid to those residents who have a longer eating time. As nurses and nursing assistants are the major caregivers of residents in long-term care facilities, it is important to connect them with residents and educate them to observe food intake problems and accordingly provide suitable assistance.

At dinnertime, food intake difficulties were negatively correlated with the illuminance level and were positively correlated with the sound volume level. These were not correlated with food intake difficulties at lunchtime. A lower illuminance level (AOR = 0.997, 95% CI: 0.995~0.998) and higher sound volume level (AOR = 1.12, 95% CI: 1.04~1.21) may be related to a higher level of food intake difficulties. In addition to environmental factors, including the illuminance and sound volume levels, cognitive function was also correlated with food intake difficulties at dinnertime. The dining environment was reported to be an important factor involved in the food intake process [42]. Moreover, cognitive impairment, such as short-term memory deficits, interferes with eating, and causes the patient to forget the task at hand or become easily distracted due to environmental factors. Possible reasons for the influence of environmental factors and cognitive function on food intake difficulties among residents with dementia at dinnertime, but not lunchtime, might have been due to sundowning syndrome of residents with dementia. Sundowning syndrome is a display of neuropsychiatric symptoms in the late afternoon, evening, or at night and is reported to be associated with impaired circadian rhythmicity, environmental and social factors, and impaired cognition [20]. A brighter and quieter dining environment might minimize residents with dementia being distracted by environmental factors and alleviate their sundowning syndrome [5,37]. According to our observations, more than one-third of illuminance levels did not meet the criteria recommended Chinese National Standards (CNS) in Taiwan [43], based on 213 participants’ mealtime observations at both lunch and dinner in this study. In addition, a television or radio is often turned on during mealtimes in long-term care facilities in Taiwan. A television was turned on at approximately 70% of mealtimes; while a radio was turned on approximately 30% of mealtimes, based on 213 participants’ mealtime observations at both lunch and dinner in this study. Therefore, it is easy to decrease the dining environmental sound volume for residents with dementia; both televisions and radios should be turned off, or residents with dementia should have a private room while dining. Enhancing the quality of meals might influence mealtime outcomes for residents with dementia [44]. According to our findings, staff members in long-term care facilities may consider changing the dining environment to create a more comfortable atmosphere during mealtime, such as increasing the amount of lighting in the evening hours, reducing overall noise levels, and providing soft instrumental music.

## Limitations

Results of this study need to be interpreted with caution as the convenience and small sample size may limit the generalizability of these findings. Further, the cross-sectional design may limit the ability to assess food intake difficulties over time and see patterns of change among residents with dementia. Stratified randomization should be applied across Taiwan in the future instead of selecting long-term care facilities in northern Taiwan in order to avoid selection bias, if the budget and the time are ample. In addition, breakfast in Chinese and Taiwanese culture is not as formal as lunch and dinner, and thus we only assessed lunch and dinner observations in this study. For example, a lot of Chinese and Taiwanese only drink soymilk for breakfast; therefore, the quantity and quality of breakfast are not comparable to those of lunch and dinner. Lastly, charts among long-term care facilities in Taiwan are not as informative as medical charts in acute-care hospitals, as they only record the diagnosis of dementia but not the specific type of dementia.

## Supporting information

S1 File (XLS)
